# Pacifier use in preterm infants during orogastric tube feeding: a randomized trial

**DOI:** 10.1590/1806-9282.20252089

**Published:** 2026-07-10

**Authors:** Aysel Topan, Meltem Kürtüncü, Sadrettin Ekmen, Yeliz Taşdelen, Derya Yiğit, Kübra Ilgaz

**Affiliations:** 1 Zonguldak Bülent Ecevit University, Faculty of Health Sciences, Department of Pediatric Nursing – Zonguldak, Türkiye.; 2 Karabük University, Faculty of Health Sciences, Department of Pediatric Nursing – Karabük, Türkiye.; 3 Karabük University, Faculty of Health Sciences, Department of Pediatric Nursing – Karabük, Türkiye.; 4 Necip Fazıl City Hospital Gynecology and Obstetrics Additional Service Building, Neonatal Intensive Care Unit – Kahramanmaraş, Türkiye.; 5 Karabuk University Karabuk Training and Research Hospital, Neonatal Intensive Care Unit – Karabük, Türkiye.

**Keywords:** Infant, Pacifiers, Tube feeding, Vital signs, Neonatal nursing

## Abstract

**OBJECTIVE:**

The aim of this study was to examine the effects of pacifier use alone and combined with human milk on vital signs, feeding duration, and gastric residue in preterm infants during orogastric tube feeding.

**METHODS:**

In total, 90 preterm infants (gestational age 29+0 to 34+0 weeks) were randomly assigned to three groups: pacifier group, pacifier with human milk group, and control group. During feeding, the pacifier group received only a pacifier, while the pacifier with human milk group received a pacifier with 1–2 mL of human milk. The control group received no intervention.

**RESULTS:**

Groups were homogeneously distributed based on descriptive characteristics. Feeding duration was significantly shorter in both intervention groups compared to the control group. Additionally, heart and respiratory rates measured after feeding were significantly lower in these groups than during feeding.

**CONCLUSION:**

These findings suggest that pacifier use, with or without human milk, may be associated with more stable physiological responses after feeding and more efficient feeding in preterm infants.

**Clinical Trial Registration Number:**

This study was prospectively registered at ClinicalTrials.gov, registration NCT05530733.

## INTRODUCTION

Over the past two decades, improved survival of preterm infants has made oral feeding a major challenge^1^. Immature neurodevelopment often prevents coordination of sucking, swallowing, and breathing, requiring initial orogastric or nasogastric tube feeding^2^. Early transition to oral feeding is essential, as tube-feeding experiences shape later feeding skills^1,2^.

Supporting a positive sucking–swallowing association is recommended to prevent feeding aversion^3^. Oral feeding demands complex coordination and can affect heart rate, respiratory rate, and oxygen saturation^3,4^. The World Health Organization (WHO) identifies lactation as the gold standard under the Baby-Friendly Hospital Initiative^5^. However, recent studies show the benefits of pacifier use on feeding behavior and physiological stability^1,6^. Although generally discouraged in Türkiye per WHO guidance, Step 9 of the NICU “Ten Steps to Successful Breastfeeding” allows pacifiers for justified reasons^7^, particularly for tube-fed infants^8^.

Pacifiers offer simple non-nutritive sucking and support oral stimulation^6^. Evidence indicates they improve feeding efficiency, stabilize vital signs, shorten tube-feeding duration, accelerate oral-feeding transition, promote weight gain, and may reduce hospital stay^4^. A meta-analysis also reported benefits during gavage feeding, including enhanced sucking behavior and digestion^9^. Pacifiers may further stimulate digestive enzymes and hormones via vagal activation^3^. Feeding intolerance remains common in preterm infants, often leading to intravenous nutrition and prolonged hospitalization. Although sensory cues like smell and taste aid digestion, tube-fed infants receive minimal exposure^2^. Exposure to the odor of human milk has been reported to exert beneficial effects on multiple parameters in preterm infants, including reductions in cortisol levels, improvements in oxygen saturation, and a shorter time to transition to oral feeding^10,11,12^. Moreover, considering that the senses of smell and taste play a critical role in activating physiological processes that contribute to digestion and nutrient absorption, tube feeding in preterm infants limits the interaction of these sensory pathways with milk, as the milk does not pass through the nasal and oral cavities during feeding^2,13^. In this context, combining non-nutritive sucking with exposure to human milk may provide simultaneous oral-motor and sensory stimulation. Using a pacifier dipped in human milk allows infants to experience both the act of sucking and the taste and smell of milk, potentially offering a more integrated sensory experience than isolated exposure.

Given the positive effects of pacifier use and the smell of human milk, this study aimed to examine how offering only a pacifier or a pacifier with human milk affects vital signs, feeding duration, and gastric residue during orogastric feeding.

Study Hypotheses: Compared to the control group, during orogastric tube feeding in preterm infants:

H0: The interventions of offering a pacifier or a pacifier with human milk have no effect on the infants’ vital signs and feeding performance.
H1a: Offering only a pacifier has an effect on lowering the infants’ heart rate.
H1b: Offering only a pacifier has an effect on reducing the infants’ respiratory rate.
H1c: Offering only a pacifier has an effect on increasing the infants’ oxygen saturation levels.
H1d: Offering only a pacifier has an effect on reducing the amount of gastric residue in the infants.
H1e: Offering a pacifier with human milk has an effect on lowering the infants’ heart rate.
H1f: Offering a pacifier with human milk has an effect on reducing the infants’ respiratory rate.
H1g: Offering a pacifier with human milk has an effect on increasing the infants’ oxygen saturation levels.
H1h: Offering a pacifier with human milk has an effect on reducing the amount of gastric residue in the infants.

## METHODS

### Design and setting

This study employed a three-group randomized controlled experimental design to compare the effects of pacifier use alone with human milk on the vital signs, feeding duration, and gastric residual volume of preterm infants during orogastric tube feeding. The study was conducted in the NICU of Karabük Education and Research Hospital between June 2022 and February 2024. The study’s reporting followed the CONSORT guidelines^14^ (Clinical trial number: NCT05530733).

### Participants and randomization

The study included premature infants treated in the NICU of Karabük Education and Research Hospital. Eligible infants were those fed via an orogastric tube, receiving both formula and human milk (with formula comprising >50% of total daily intake), born between 29+0 and 34+0 weeks of gestation, and weighing ≥1,000 g at enrollment. Infants requiring mechanical ventilation, diagnosed with sepsis, neurological diseases, chromosomal or congenital anomalies, intraventricular hemorrhage, showing unstable physiological parameters, or receiving no human milk were excluded.

As no prior similar studies were available, sample size estimation was based on a pilot study with 15 infants (pacifier: n=5; pacifier with human milk: n=5; control: n=5). Using pilot data, the effect size was calculated as 0.35 with G*Power 3.1.9.4. With α=0.05 and power=0.80, the minimum required sample size was 81. To allow for potential dropouts, 90 infants were included (30 per group).

**Figure 1:**
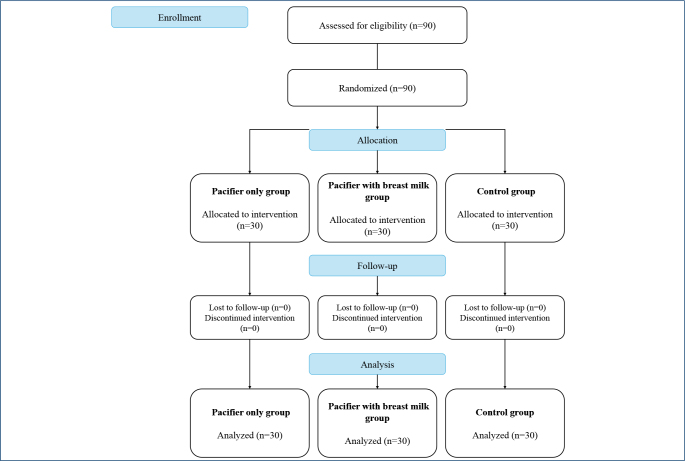
CONSORT flow diagram of the study participants.

Infants were allocated to groups via simple randomization using a Microsoft Excel random number generator. Due to the intervention’s nature, the practitioner delivering the intervention and measurements was not blinded; however, the data analyst was blinded to group assignments. The study’s CONSORT flow diagram is shown in Figure 1.

### Data collection forms and tools

Participant Information Form: This form, containing nine questions, was designed by the researchers based on the literature^1,9,15^ and includes the sociodemographic characteristics of the infants.

Premature Infant Follow-up Form: This form was created to record data collected during the orogastric tube feeding of preterm infants. It includes information about the group to which the infant was assigned (pacifier group, pacifier with human milk group, or control group), feeding duration, formula intake, gastric residue volume, number of days fed via orogastric tube, and the infant’s vital signs.

Feeding duration was defined as the total time required to deliver the prescribed feeding via an OG tube. During feeding, the prepared formula was drawn into a feeding syringe and connected to the OG tube. The feeding time was initiated at the moment the syringe was connected to the OG tube. After connection, the syringe plunger was removed, allowing the milk to be administered by gravity under ambient air pressure through the OG tube. The feeding duration was stopped when the syringe was completely emptied. Feeding duration was measured using this standardized procedure for all study groups. This standardized and predefined procedure was applied to minimize operator-related variability and potential measurement bias. In the study institution, there is no standardized protocol regarding feeding duration.

Pacifier: Each infant in the study was provided with a Babysoft brand silicone pacifier for premature infants. The pacifiers were sterilized before use.

### Interventions

#### Pacifier group

Infants in this group received 100 mL/kg/day of formula via orogastric tube during a single feeding session. A pacifier was given 5 min before feeding and kept in place throughout the feeding and for 5 min afterward. Vital signs (heart rate, respiratory rate, and oxygen saturation) were recorded from the bedside electronic monitor before, during, and after feeding and documented on the Premature Infant Follow-up Form. Gastric residue was assessed 2 h post-feeding.

#### Pacifier with human milk group

These infants followed the same feeding protocol; however, the pacifier was moistened with 1–2 mL of the infant’s mother’s milk and given 5 min prior to feeding. Vital signs were recorded from the bedside electronic monitor before, during, and after feeding in the same manner. Gastric residue was measured 2 h after feeding.

#### Control group

Control infants received routine care and were fed 100 mL/kg/day of formula via orogastric tube. Vital signs were obtained from the bedside electronic monitor 5 min before feeding, during feeding, and 5 min after feeding. Gastric residue was evaluated 2 h later.

### Data collection

Parents of eligible infants were informed about the study, and written consent was obtained from mothers. In the NICU, infants’ heart rate, oxygen saturation, and blood pressure were continuously monitored, and they were fed via orogastric tube until they developed an adequate sucking reflex. Pacifiers were not routinely used during tube feeding. After randomization, infants were assigned to one of the three groups. The Participant Information Form was completed, and if infants were receiving 100 mL/kg/day via orogastric tube per physician orders, the intervention was applied during one scheduled feeding session at 14:00 using standard premature infant pacifiers.

### Statistical analysis

Data were analyzed using IBM SPSS version 24.0 (IBM SPSS Corp., Armonk, NY, USA). Descriptive characteristics were summarized with frequencies, percentages, means, and the chi-square test. Normality was assessed using the Kolmogorov-Smirnov test along with skewness–kurtosis values and histogram inspection. Normally distributed data were analyzed with mean±standard deviation and parametric tests, while non-normally distributed data were evaluated with median (Q1–Q3) and non-parametric tests. Group differences were examined using Pearson chi-square, Fisher exact, Kruskal-Wallis, and one-way ANOVA tests. For repeated measures within groups, Friedman and Wilcoxon signed ranks tests were applied. Posthoc analyses were conducted to determine the source of significant differences. When the Kruskal-Wallis test was applied, post-hoc pairwise comparisons were performed using the Mann-Whitney U test with Bonferroni correction. Only statistically significant comparisons are presented in the tables. Statistical significance was set at p<0.05 with a 95%CI.

### Ethical considerations

Ethical approval was obtained from the Zonguldak Bülent Ecevit University Non-Interventional Clinical Research Ethics Committee (Protocol no: 2022/10, Date: April 2022), and written permission was secured from Karabük Education and Research Hospital (Protocol no: E-34771223-774.99, Date: April 2022), where the study was conducted. Participant recruitment commenced in June 2022, following ethical approval. Informed consent was provided to participants, explaining the study’s purpose, data confidentiality, voluntary participation, and the right to withdraw at any time.

## RESULTS

No significant differences were found between the groups (pacifier-only, pacifier with human milk, and control) in terms of descriptive characteristics (p>0.05). Gastric residual volume was minimal and showed no variability across all groups, precluding any meaningful statistical comparison or clinical interpretation (Table 1).

**Table 1:** Comparison of descriptive characteristics of preterm infants.

Variables	Pacifier only (n=30)	Pacifier with human milk (n=30)	Control (n=30)	Test (df), p
Gender, n (%)				
Girl	15 (50.0)	16 (53.3)	15 (50.0)	0.089 (2) p=1.000^a^
Boy	15 (50.0)	14 (46.7)	15 (50.0)	
Type of delivery, n (%)				
Vaginal	1 (3.3)	2 (6.7)	2 (6.7)	0.609 (2) p=1.000^b^
Cesarean section	29 (96.7)	28 (93.3)	28 (93.3)	
Multiple pregnancy, n (%)				
Yes	10 (33.3)	9 (30.0)	9 (30.0)	0.104 (2) p=1.000^a^
No	20 (66.7)	21 (70.0)	21 (70.0)	
Gestational age (week), median (Q1–Q3)	32.78 (31.64–33.46)	32.35 (31.10–34.00)	32.42 (31.35–34.00)	0.044 (2) p=0.763^c^
Postnatal age (week), median (Q1–Q3)	1.50 (0.85–3.57)	1.64 (0.96–2.71)	1.50 (0.85–2.64)	0.447 (2) p=.800^c^
Birth length (cm), median (Q1–Q3)	42.00 (39.50–44.00)	42.50 (41.00–46.00)	42.00 (40.75–46.25)	1.070 (2) p=0.586^c^
Birth weight (g), mean±SD	1,888.00±478.44	1,854.50±442.04	1,911.83±542.06	0.104 (2) p=0.901^d^
Current weight (g), median (Q1–Q3)	1,972.50 (1,751.25–2,146.25)	2,022.50 (1,763.75–2,232.50)	1,960.00 (1,728.75–2,231.25)	0.101 (2) p=0.951^c^
Days on mechanical ventilation, median (Q1–Q3)	3.50 (1.00–10.25)	5 (2.00–8.50)	4.50 (2.00–10.00)	1.682 (2) p=0.431^c^
Amount of formula intake, mL, median (Q1–Q3)	32.50 (30.00–43.00)	35.00 (30.00–43.25)	35.00 (30.00–40.75)	1.784 (2) p=0.410^c^
Days of feeding with an OG, median (Q1–Q3)	9.00 (5.00–19.25)	11.00 (5.00–17.75)	8.50 (5.00–17.50)	0.575 (2) p=0.750^c^
Gastric residual volume, mL	0.00 (0.00–0.00)	00.00 (0.00–0.00)	0.00 (0.00–0.27)	5.105 (2) p=0.07^c^

cm: centimeter; g: gram; mL: milliliter; OG: orogastric; df: degrees of freedom;

^a^Pearson Chi-square Test;

^b^Fisher Exact Test;

^c^Kruskal-Wallis Test;

^d^One-way ANOVA.

Both the pacifier and pacifier with human milk groups had statistically significantly shorter feeding times than the control group (adjusted p<0.001). There were no statistically significant differences in heart rate, respiratory rate, or oxygen saturation levels between the groups before, during, or after feeding (p>0.05). Post-feeding heart rates were significantly lower in the pacifier (p<0.001) and pacifier with human milk (p=0.03) groups than those measured during feeding. In the control group, no significant difference in heart rate was found between during and after feeding (p>0.05). In both the pacifier (p<0.001) and pacifier with human milk (p=0.01) groups, the respiratory rate measured after feeding was statistically significantly lower than that during feeding. No significant differences were found in respiratory rates during and after feeding in the control group (p>0.05). Oxygen saturation values did not differ significantly between groups before, during, or after feeding (p<0.05) (Table 2).

**Table 2:** Comparison of feeding duration, gastric residual volume, and vital signs of the preterm infants.

Measurement time	Pacifier only (n=30)	Pacifier with human milk (n=30)	Control (n=30)	Test (df), p
	Median (Q1–Q3)	Median (Q1–Q3)	Median (Q1–Q3)	
Feeding duration, min	10.00 (9.00–12.00)	10.00 (9.00–11.25)	12.00 (11.75–14.00)	**22.790 (2)** **p<0.001^c^** **1<3** **2<3**
Heart rate, min				
Before feeding (1)	146.50 (141.50–155.25)	148.00 (141.50–156.00)	146.50 (139.00–154.00)	0.330 (2) p=0.848^c^
During feeding (2)	156.00 (148.00–160.00)	154.00 (149.25–160.00)	152.00 (145.50–157.25)	2.881 (2) p=0.245^c^
After feeding (3)	150.00 (143.50–154.00)	151.50 (143.75–154.00)	152.00 (142.00–154.25)	0.877 (2) p=0.645^c^
Test, p	**27.239 (2)** **p<0.001^e^** **2>1, 2>3**	**11.241 (2)** **p=0.004^e^** **2>1, 2>3**	**12.776** **p=0.002^e^** **2>1**	
During–after feeding difference	-6.50 (-12.00 to -4.00)	-4.00 (-8.50–2.00)	-2.00 (-4.00–2.00)	
Test, p	**-4.162** **p<0.001^f^**	**-2.166** **p=0.03^f^**	-1.852 p=0.06^f^	
Respiration rate, min				
Before feeding (1)	52.00 (50.00–54.00)	50.00 (50.00–52.50)	52.00 (50.00–53.25)	0.738 (2) p=0.692^c^
During feeding (2)	55.00 (52.00–56.50)	52.00 (52.00–56.00)	52.00 (52.00–54.50)	2.858 (2) p=0.240^c^
After feeding (3)	52.00 (50.00–54.00)	51.00 (50.00–54.00)	52.00 (51.00–54.00)	3.133 (2) p=0.209^c^
Test, p	**23.842 (2)** **p<0.001^e^** **2>1, 2>3**	**14.373 (2)** **p<0.001^e^** **2>1, 2>3**	**20.609 (2)** **p<0.001^e^** **3>1, 2>1**	
During–after feeding difference	-2.00 (-4.50 to -0.75)	-2.00 (-4.00–0.50)	0.00 (-2.00–0.00)	
Test, p	**-3.479** **p<0.001^f^**	**-2.455** **p=0.01^f^**	-1.851 p=0.06^f^	
Oxygen saturation, %				
Before feeding (1)	98.00 (96.00–99.00)	97.00 (95.75–98.00)	98.00 (96.75–98.00)	4.476 (2) p=0.107^c^
During feeding (2)	97.00 (96.00–99.00)	97.00 (95.00–98.00)	98.00 (96.75–98.25)	1.940 (2) p=0.379^c^
After feeding (3)	98.00 (96.00–99.00)	97.00 (95.00–98.00)	98.00 (97.00–98.00)	1.969 (2) p=0.374^c^
Test, p	1.156 (2) p=0.561^e^	4.066 (2) p=0.131^e^	1.080 (2) p=0.583^e^	
During–after feeding difference	0.00 (0.00–1.00)	0.00 (0.00–1.00)	0.00 (0.00–0.00)	
Test, p	0.586 p=0.558^f^	0.676 p=0.499^f^	-0.165 p=0.869^f^	

min: minute; mL: milliliter; df: degrees of freedom;

^c^Kruskal-Wallis Test;

^e^Friedman Test;

^f^Wilcoxon signed ranks test. Bold values indicate statistical significance (p<0.05).

## DISCUSSION

Our study demonstrated several benefits of pacifier use in premature infants fed via orogastric tube, particularly the reduction in feeding duration in both intervention groups compared with the control group. Although the literature lacks direct evidence on pacifier or human milk–soaked pacifier use during orogastric feeding, studies by Calik and Esenay^6^ reported earlier oral feeding, improved sucking skills, greater weight gain, and shorter hospital stays in infants using pacifiers. Meta-analytic findings further support the positive impact of non-nutritive sucking on the transition to full oral feeding, intestinal transit time, and hospitalization length^3^. Exposure to smell and taste, which facilitate digestive activation, is limited during tube feeding. Evidence on the effects of milk odor or taste remains inconsistent, with some studies reporting unclear findings^2,13^. However, one meta-analysis noted that human milk odor shortened the transition to oral feeding and reduced parenteral nutrition duration, though it did not influence hospital stay^12^. Based on existing evidence, we suggest that pacifier use during orogastric feeding may support sucking–swallowing coordination and help shorten feeding time. The absence of a difference between the pacifier-only and pacifier with human milk groups may be related to the single-session evaluation. While milk odor or taste has shown positive effects during painful procedures, further research is needed to clarify the additional benefits of combining pacifier use with human milk^16^.

Our study showed that pacifier use (with or without human milk) reduced post-feeding heart and respiratory rates, whereas no such change was seen in the control group. Prior evidence similarly indicates that non-nutritive sucking does not adversely affect physiological parameters^3^ and improves physiological and behavioral responses in infants^4,17^. Pacifiers are also preferred by parents for their calming effect, and the American Academy of Pediatrics recommends their use during sleep to reduce Sudden Infant Death Syndrome (SIDS) risk^18^ Human milk odor or taste has been associated with improved physiological stability, including reduced cortisol and increased oxygen saturation during procedures^10^ and enhanced digestive function^11^. In this context, pacifier use may help regulate physiological responses, and human milk may offer additional benefits. The observed decreases in heart and respiratory rates after feeding in the intervention groups likely indicate a return to baseline, more stable physiological states rather than a reduction below normal levels. This stabilization may enhance feeding tolerance and reduce stress during orogastric feeding, potentially facilitating smoother transitions to oral feeding. Although these findings suggest potential physiological benefits, further studies are warranted to evaluate long-term outcomes and individualized approaches to pacifier use in preterm infants. However, our study found no effect on oxygen saturation, possibly because all infants tolerated ≥50% of their nutritional needs and had no respiratory problems.

Regarding feeding tolerance, gastric residual volumes did not differ among the groups. Although non-nutritive sucking has been linked to reduced feeding intolerance and fewer gastrointestinal symptoms^1,19^, and is believed to support rhythmic sucking–swallowing patterns and gastrointestinal maturation^19^, our findings did not demonstrate a between-group difference in gastric residual volume. However, the almost universal absence of gastric residuals and the limited variability observed across groups suggest a possible floor effect. Therefore, these findings should be interpreted with caution, as the study population consisted of clinically stable infants with good feeding tolerance, and although the study was adequately powered for the feeding duration, the very low variability and floor effect in gastric residual volume likely reduced the statistical power to detect meaningful differences for this outcome.

### Strengths and limitations

A strength of this design is the comparative evaluation of pacifier use and pacifier use with human milk in three groups of infants fed via an orogastric tube. This allowed for a comparison of interventions on heart and respiratory rates, contributing to evidence on non-nutritive sucking and feeding coordination.

The main limitation is the single-session assessment, which restricts the evaluation of long-term effects. Repeated or longitudinal measurements may reveal outcomes not captured here. Studies should include longer follow-up to improve generalizability. The limited variability in gastric residual volume represents another limitation of this study and may have reduced the statistical power to detect group differences. This floor effect, with most gastric residual volume values being zero, limited the ability to detect potential between-group differences in feeding tolerance. In addition, blinding of the care provider was not feasible due to the nature of the intervention. During feeding and outcome measurements, the practitioner was aware of the infant’s group allocation, which may have introduced performance and detection bias. Another limitation of this study is that the effect size used for the sample size calculation was derived from a small pilot study (n=15), which may have affected the precision of the power estimation. Finally, the study population consisted of relatively mature preterm infants who were receiving more than 50% formula feeding. This may limit the generalizability of the findings to more fragile or very preterm infants, as well as to populations exclusively fed with human milk.

## CONCLUSION

Feeding durations were shorter in both intervention groups, while gastric residual volumes were similar across groups. In the pacifier and pacifier with human milk groups, decreases in heart and respiratory rates were observed, reflecting a return to baseline physiological states and suggesting potential clinical benefits for feeding tolerance. These findings support pacifier use during orogastric feeding and transition to oral feeding. Research should examine long-term effects of pacifier and human-milk-enhanced pacifier use, and individualized strategies considering pacifier-related physiological responses may optimize feeding and support oral-feeding transitions.

## Data Availability

The datasets generated and/or analyzed during the current study are available from the corresponding author upon reasonable request.
